# Epigenetics and airways disease

**DOI:** 10.1186/1465-9921-7-21

**Published:** 2006-02-06

**Authors:** Ian M Adcock, Paul Ford, Kazuhiro Ito, P J Barnes

**Affiliations:** 1 Airways Disease Section, National Heart and Lung Institute, Imperial College London, UK

## Abstract

Epigenetics is the term used to describe heritable changes in gene expression that are not coded in the DNA sequence itself but by post-translational modifications in DNA and histone proteins. These modifications include histone acetylation, methylation, ubiquitination, sumoylation and phosphorylation. Epigenetic regulation is not only critical for generating diversity of cell types during mammalian development, but it is also important for maintaining the stability and integrity of the expression profiles of different cell types. Until recently, the study of human disease has focused on genetic mechanisms rather than on non-coding events. However, it is becoming increasingly clear that disruption of epigenetic processes can lead to several major pathologies, including cancer, syndromes involving chromosomal instabilities, and mental retardation. Furthermore, the expression and activity of enzymes that regulate these epigenetic modifications have been reported to be abnormal in the airways of patients with respiratory disease. The development of new diagnostic tools might reveal other diseases that are caused by epigenetic alterations. These changes, despite being heritable and stably maintained, are also potentially reversible and there is scope for the development of 'epigenetic therapies' for disease.

## Introduction

The genetic code cannot be the sole arbiter of cell fate since each cell in a blastocyst can differentiate into the many different cell types found in multicellular organisms each with a unique function and gene expression pattern. This has led to the idea that additional information beyond that generated by the genetic code must be important for the regulation of genomic expression. Over 60 years ago the term "epigenetics" was introduced to describe this information and this is now understood to mean all meiotically and mitotically heritable changes in gene expression that are not coded in the DNA sequence itself [1]. Epigenetic regulation is not only critical for generating diversity of cell types during mammalian development, but it is also important for maintaining the stability and integrity of the expression profiles of different cell types. Interestingly, whereas these epigenetic changes are heritable and normally stably maintained, they are also potentially reversible, as evidenced by the success of cloning entire organisms by nuclear transfer methods using nuclei of differentiated cells [2]. Therefore, understanding the basic mechanisms that mediate epigenetic regulation is invaluable to our knowledge of cellular differentiation and genome programming.

Studies of the molecular basis of epigenetics have largely focused on mechanisms such as DNA methylation and chromatin modifications [3]. In fact, emerging evidence indicates that both mechanisms act in concert to provide stable and heritable silencing in higher eukaryotic genomes. Interestingly, the recently described process of RNA silencing, originally utilised by the cell to protect itself against viral infection, also involves the same mechanisms used to sustain epigenetic silencing. These components (DNA methylation, chromatin modifications and RNA-associated silencing) interact and often disruption of one component will affect the activity/expression of the other two leading to inappropriate expression or silencing of genes, resulting in 'epigenetic diseases' [1,3].

It is possible for epigenetic marks to be transmitted along chromosomes. *Drosophila *and plants exhibit a characteristic known as position-effect variegation (PEV) whereby euchromatic genes can become transcriptionally silenced when juxtaposed to heterochromatic sequences [1]. The extent of this cis-spreading silencing phenomenon varies and involves a number of proteins which have roles in heterochromatin formation e.g. E(var)s (enhancers of PEV) or Su(var)s (suppressors of PEV) [4]. Su(var) 2–5 for example encodes the chromatin-binding nuclear protein heterochromatin protein 1 (HP1) [5] which has a critical role in initiating and maintaining the condensed chromatin conformation of heterochromatin through its actions on histone methylation and chromatin remodelling.

### Epigenetic marks

#### DNA methylation

One of the most fundamental epigenetic marks is the widespread methylation of the C^5 ^position of cytosine residues in DNA [1,6]. The maintenance of these methyl CpG marks is due to the action of a number of DNA methyltransferases (DNMTs) which add the universal methyl donor S-adenosyl-L-methionine to cytosine (Table 1). These enzymes have been implicated in many processes including transcriptional regulation, genomic stability, chromatin structure modulation, X chromosome inactivation, and the silencing of parasitic DNA transposable elements [7]. Overall, DNA methylation exerts a stabilizing effect which promotes genomic integrity and ensures proper temporal and spatial gene expression during development. In contrast, DNA demethylation is probably a passive event and no *bona fide *DNA demthylase has been identified to-date [8]. The importance of DNA methylation is highlighted by the fact that many human disease result from abnormal control [9]. In addition, cytosine methylation is highly mutagenic, causing a C to T mutation resulting in loss of the CpG methyl-acceptor site, and aberrant methylation of CpG islands is a characteristic of many human cancers and may be found in early carcinogenesis [3,10,11].

It has been estimated that as much as 80% of all CpG dinucleotides in the mammalian genome are methylated [1]. The remaining unmethylated CpG residues are mostly located in the promoter regions of constitutively active and/or inducible genes and are referred to as CpG islands. CpG islands generally consist of regions of >500 base pairs with a GC content greater than 55% [9,12]. When methylated these CpG islands result in stable inherited transcriptional silencing. How sequences are targeted for *de novo *methylation in mammals remains largely unknown. Several triggers have been proposed to target DNA methylation including: (i) sequence, composition or secondary structure of the DNA itself; (ii) RNAs that might target regions on the basis of sequence homology; and (iii) specific chromatin proteins, histone modifications or higher-order chromatin structures and these are clearly not mutually exclusive [13].

**Table 1:** DNA methyltransferases (DNMTs) and methyl binding proteins. Dnmts establish and maintain methylation marks whilst methyl CpG binding proteins interpret these marks.

**DNA methyltransferase**	**Activity**	**Function**
DNMT1	Prefers hemi-methylated DNA	Maintenance of methylation, repression of transcription
DNMT2	Low activity *in vitro*	Non CpG methylation in Drosphilia
DNMT3a	*De novo *methylation	Imprinting and repression
DNMT3b	*De novo *and maintenance methylation	Repeat methylation, repression
DNMT3L	Not active, co-localizes with DNMT3a and 3b	Repeat methylation, repression
**Methyl CpG binding protein**	**Specificity**	
		
MeCP2	Single methylated CpG	Repression
MBD1	Methylated and unmethylated DNA	Repression
MBD2	Methylated DNA	Repression
MBD3	Unmethylated DNA	Repression
MBD4	^5-*me*^CpG/TpG mismatches	DNA repair,

Abbreviations: MeCP – Methyl-CpG-binding protein, MBD – methyl-CpG binding domain.

Early models for the control of DNA methylation proposed two-steps: '*de novo *methylation' by DNMTs active on unmethylated DNA e.g. DNMT3a and 3b [14], followed by 'maintenance methylation' by DNMT3a or by DNMT1 which is specific for the hemi-methylated DNA resulting from replication [15]. However, the validity of this model has recently been questioned [9]. There are a number of DNMTs and DNMT-interacting proteins reported mostly distinguished on the basis of structural similarity, sequence specificity but rarely primary function. Indeed most predicted proteins have been designated as being DNMTs solely because they have most, or all, of the conserved motifs observed in the catalytic domain of known DNMTs [9,10]. The problem is compounded by the fact that DNMTs may also form complexes with each other [16].

Mammalian Dnmt1 is considered to be a maintenance DNMT as knockout studies and antisense approaches show a global effect on methylation [9,17]. Furthermore, DIM-2, a relative of Dnmt1, is responsible for all known DNA methylation in *Neurospora *[13]. Some potential DNMTs include proteins for which little or no enzymatic activity has been found in mammalian cells [13], thus, mammalian DNMT2 has little or no DNMT activity *in vitro *[18], and deletion of *Dnmt2 *in mouse embryonic stem cells had no noticeable effect on DNA methylation [13]. In contrast, depletion of *Drosophila Dnmt2 *by RNAi, however, resulted in loss of the little DNA methylation detectable by immunolocalization, and overexpression appeared to induce hypermethylation [19].

DNA methylation can repress transcription through several mechanisms including direct inhibition of transcription factor DNA binding and indirectly through the effects of methyl CpG binding proteins (Table 1). As such, methyl-CpG binding proteins e.g. MeCP2 and MBDs are recruited to methylated CpG where they can act as mediators of transcriptional repression through the association with HDAC containing repressor complexes. Interestingly, *Mbd2 *knockout cells can express IL-4 in cells where this gene is normally silent [20]. In contrast, CpG methylation blocks DNA binding of the chromatin boundary element binding protein (CTCF), which can block interactions between an enhancer and its promoter when placed between the two elements resulting in gene induction. Generally loss of MBDs is less profound than that of DNMT loss since DNMTs greatly reduce the extent of genomic DNA methylation and therefore interfere with all proteins that interpret the DNA methylation signal whereas loss of one methyl-CpG binding protein will enable other proteins that recognize the DNA methylation signal.

DNA methylation, in conjunction with post-translational modifications of histones, is involved in the regulation of chromatin states that are either mutually reinforcing or mutually inhibitory possibly acting through feedback loops [17]. This may polarize chromatin, committing it to enable either transcriptional activity or transcriptional silence with uncommitted states being rare. This would imply that an active mechanism must be involved in switching between transcriptionally active and silenced states. Recently, clear evidence for cross-talk between these epigenetic processes has been provided. Thus, the polycomb group (PcG) protein EZH2 (Enhancer of Zeste homolog 2) serves as a recruitment platform for DNMTs indicating a direct link between the two major epigenetic repression systems [21]. Similarly, histone H1 depletion induced marked changes in chromatin structure such as decreasing global nucleosome spacing and reducing local chromatin compaction without affecting global DNA methylation. However, many of the genes whose expression was regulated by H1 depletion showed evidence for reduced methylation of specific CpGs within their regulatory regions thereby suggesting that linker histones can also play a role in the maintenance or establishment of specific DNA methylation patterns [22].

### Chromatin structure and histone modifications

Chromatin is made up of nucleosomes which are particles consisting of 146 bp of DNA wrapped around an octomer of two molecules each of the core histone proteins (H2A, H2B, H4 and H4). Nucleosomal DNA can be further compacted by association with the linker histone H1 and additional nonhistone proteins, as well as by higher order looping and folding of the chromatin fibre. In the resting cell DNA is wound tightly around these basic core histones, presenting an impenetrable barrier to large protein complexes such as RNA polymerase II, which produce unspliced primary messenger RNA transcripts. Alterations in the structure of chromatin are critical to the regulation of gene expression [1,23,24].

**Figure 1:**
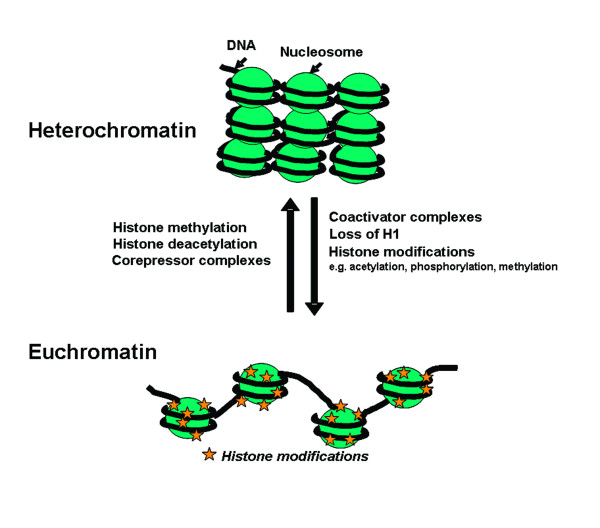
Heterochromatin is the compacted "closed" form of chromatin associated with gene silencing. Activation of chromatin to its more "open" form which allows gene expression to occur is regulated by modification of core histones by specific co-activator complexes containing enzymes which can acetylate, phosphorylate or methylate histone tails. Removal of the linker histone H1 and changes in DNA methylation state are also important in this process. This is reversed by corepressor complexes that include histone deacetylases (HDACs) and both DNA and histone methylases, thereby causing gene silencing.

Over 100 years ago cytologists appreciated the link between chromatin compaction and cell activation status. Thus chromatin was divided into two major forms: heterochromatin and euchromatin [1]. Heterochromatin was defined as condensed regions of the nucleus that do not decondense during interphase, whereas euchromatin was noted to readily decondense upon exit of mitosis. It was postulated that heterochromatin is the functionally inactive regions of the genome and euchromatin is where gene activity occurs (Figure 1). We now know that heterochromatin regions less susceptible to nuclease activity; contain few actively expressed genes, and replicate late in the S-phase [1,25]. In contrast, euchromatin is more open and accessible to nucleases, is rich in actively transcribing genes, and replicates early during S-phase [1,25].

Allfrey and colleagues [26] initially described a role for histone acetylation in *de novo *mRNA synthesis in 1964 however it wasn't until the mid 1990s that a molecular appreciation of the events linking histone acetylation and gene expression were made. In these later studies it was reported that transcriptional co-activator proteins act as the molecular switches that control gene transcription and all have intrinsic histone acetyltransferase (HAT) activity [27,28]. Gene transcription occurs when the chromatin structure is opened up, with loosening of the tight nucleosomal structure allowing RNA polymerase II and basal transcription complexes to interact with DNA and initiate transcription. When transcription factors are activated they bind to specific recognition sequences in DNA and subsequently recruit large coactivator proteins, such as cAMP-response element binding protein (CREB)-binding protein (CBP), p300 and PCAF (p300-CBP associated factor) and other complexes to the site of gene expression [23].

**Figure 2:**
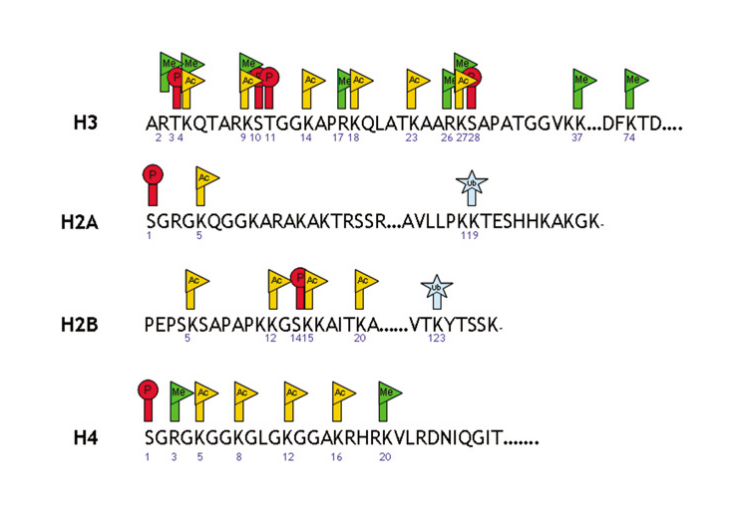
Epigenetic modifications within the nucleosomes. A number of distinct post-translational modifications including acetylation (orange flag), phosphorylation (red circle), ubiquitination (blue star) and methylation (green flag) occur at the N termini of histones H2A, H2B, H3 and H4. Other modifications are known and may also occur in the globular domain. Methylation of C5 on cytosine residues within CpG regions of DNA are also important markers for epigenetic effects. The histones are depicted in single-letter amino-acid code with the residue number shown underneath.

**Figure 3:**
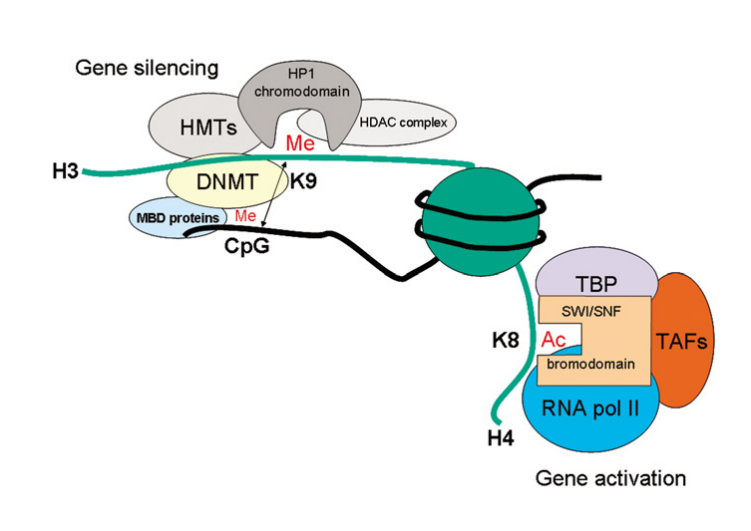
Histone modifications act by serving as a node for the assembly of coactivators and corepressor complexes through the recognition of these modifications by proteins that contain bromodomains (recognize acetylated lysines) or chromodomains (recognize methylated lysines). This, rather than an effect on chromatin structure per se, determines effects on gene expression. Recruitment of heterochromatin protein (HP)1 through a chromodomain may also affect local DNA methylation through recruitment of DNA methyltransferases (DNMTs) and methyl binding domain (MBD) proteins. This may lead to further assembly of other histone methyl transferases (HMTs) and histone deacetylase (HDAC) complexes which enable further gene silencing to occur. Gene activation, in contrast, requires recruitment of an acrivation complex involving the TATA binding protein (TBP) and its associated factors (TAFs), chromatin remodeling complexes such as mating type switching/sucrose non-fermenting (SWI/SNF) and RNA polymerase II (RNA pol II).

The N-terminal tails of the histone molecules protrude through and beyond the DNA coil presenting accessible targets for post-translational modifications such as acetylation, phosphorylation, methylation, sumoylation and ubiquitination of selective amino acid residues (Figure 2). Some modifications, including acetylation and phosphorylation, are reversible and dynamic and are often associated with inducible expression of individual genes. Thus, lysine residues in the tails of histone H3 and H4 may be acetylated forming bromodomains enabling the association of other co-activators such as TATA box binding protein (TBP), TBP-associated factors, chromatin modifying engines and RNA polymerase II [23,28](Figure 3). This molecular mechanism is common to all genes, including those involved in differentiation, proliferation and activation of cells. Just as acetylation of histones is associated with gene induction, removal of acetyl groups by histone deacetylases (HDAC)s is generally associated with re-packing of chromatin and a lack of gene expression or gene silencing [29]. Other modifications, such as methylation, are generally more stable and are involved in the long-term maintenance of expression status. Since these modifications occur on multiple but specific sites it has been suggested that modified histones can act as signalling templates, integrating upstream signalling pathways to elicit appropriate nuclear responses such as transcription activation or repression [30]. The Histone Code Hypothesis proposes that different combinations of histone modifications may result in distinct outcomes in terms of chromatin-regulated functions [31].

### Histone acetylation

Recruitment of a histone modifying enzyme to the right place at the right time is only the first step in establishing a combination of histone marks that may direct a biological outcome. The second step in this process revolves around the specificity of the enzyme for individual histone tails and for specific histone residues [23]. For example, Gcn5 (general control non-derepressible 5) and PCAF preferentially acetylate H3 K9 and K14 whereas NuA4 HAT complexes preferentially acetylate K4, K8, K12 and K16 of histone H4 [32] (Table 1).

It was originally proposed that histone acetylation would alter the electrostatic interaction between histones and DNA by altering the charge on the lysine residue leading to an "open" structure. However, at best, full acetylation of histone H3 is likely to lead to a 10–30% decrease in positive charge which is unlikely to affect interactions with DNA [32]. The major role of acetylated histones is to direct the binding of nonhistone proteins. For example, bromodomains specify binding to acetylated lysines but this does not show much specificity. For instance, acetylation of K8 within histone H4 can promote the recruitment of the ATP-dependent chromatin remodeling enzyme, human SWI/SNF – via a bromodomain within the Brg1 subunit – but a similar bromodomain within the Swi2 subunit of the yeast SWI/SNF complex interacts with a broader range of acetylated H3 and H4 tails [32,33]. Thus, the major role of the bromodomain, and the chromodomain (see later), is to serve as the nidus for assembly of co-activator vs. co-repressor complexes (Figure 3).

HATs are divided into five families. These include the Gcn5 (general control non-derepressible 5)-related acetyltransferases (GNATs); the MYST (for 'MOZ, Ybf2/Sas3, Sas2 and Tip60)-related HATs; p300/CBP HATs; the general transcription factor HATs, which include the TFIID subunit TAF250 (TBP-associated factor of 250 kDa); and the nuclear hormone-related HATs SRC1 (steroid receptor coactivator 1) and ACTR (activator of retinoid receptor) [34]. In addition to these three major groups of HATs, more than a dozen other proteins have been shown to possess acetyltransferase activity [34].

Most HATs exist as stoichiometric multisubunit complexes *in vivo *[35]. The complexes are typically more active than their respective catalytic subunits and display distinct substrate specificities [36,37], suggesting that associated subunits regulate the activities of the respective catalytic subunits. In addition, non-catalytic subunits are also involved in recruiting substrates for targeted action to ensure the specificity. One HAT can be the catalytic subunit of multiple complexes thus, GCN5L forms at least two distinct multisubunit complexes [35], and yeast Gcn5 is the catalytic subunit of four complexes [34]. Increasingly levels of complexity are being found e.g. recent studies indicate that Ubp8, a deubiquitinating enzyme present in two Gcn5 complexes, controls the deubiquitination of histone H2B and methylation of histone H3 [38]. Incorporation of HATs into complexes also alters lysine specificity. On free histones Gcn5 alone acetylates mainly H3 lysine 14, SAGA acetylates lysines 9, 14, 18 and 23, and ADA acetylates 9, 14 and 18 [35,39]. Thus, HAT complexe subunits not only specify histone modification, but also transcriptional function in targeting of these complexes to promoters.

### Histone deacetylases

**Figure 4:**
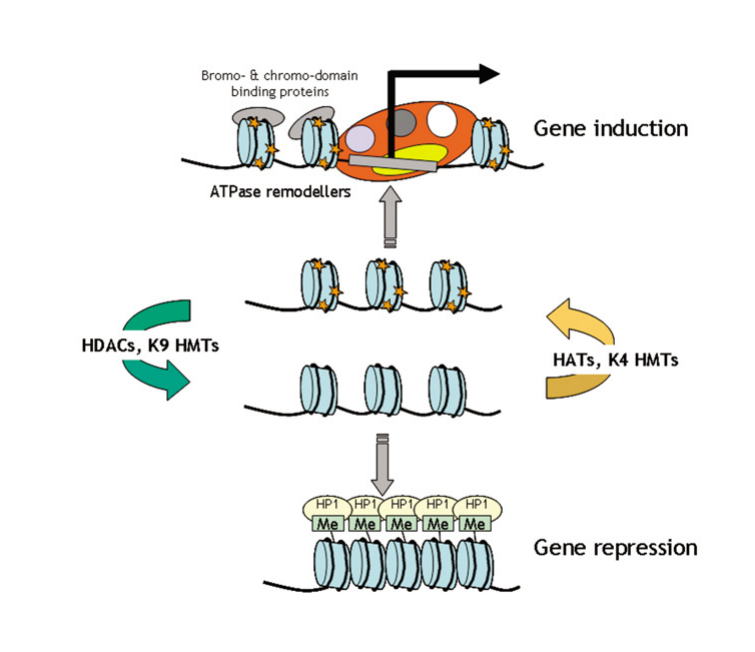
The histone switch. Targeted modifications under the control of histone methylases (HMTs), histone acetyltransferases (HATs) and histone deacetylases (HDACs) alter the histone code at gene regulatory regions. This establishes a structure that contains bromo- and chromo-domains that permits recruitment of ATP-dependent chromatin remodelling factors to open promoters and allow further recruitment of the basal transcription machinery. Deacetylation, frequently followed by histone methylation, establishes a base for highly repressive structures, such as heterochromatin. Acetylated histone tails are shown as yellow stars. Methylation (Me) is shown to recruit heterochromatin protein 1 (HP-1).

HDACs play a critical role in reversing the hyperacetylation of core histones. Lysine acetylation is reversible and is controlled by the opposing actions of HATs and HDACs *in vivo *(Figure 4). Since histones were thought to be the major cellular proteins modified by lysine acetylation, most lysine HATs and HDACs were initially identified as histone acetyltransferases and HDACs [23,40].

**Figure 5:**
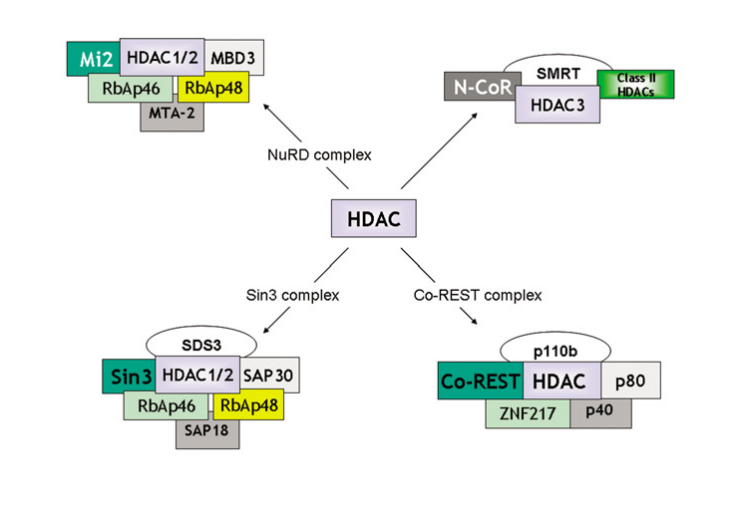
Composition of HDAC repressor complexes. HDACs lack intrinsic repressor activity and require co-factors for optimal HDAC activity. The co-repressor proteins involved in the major HDAC complexes NuRD (nucleosome remodeling and deacetylase), Sin3 (Switch insensitive 3), Co-REST (Co-repressor of REST (RE1 silencing transcription factor)) and N-CoR and SMRT complexes are shown. NuRD and sin3 complexes share the retinoblastoma associated protein (RbAp)46 and 48 proteins and also contain distinct sets of proteins. Abbreviations: Co-REST, Co-repressor of REST (RE1 silencing transcription factor); MBD3, Methyl CpG binding domain 3; Mi2, Mi2 autoantigen; MTA-2, Metastasis-associated gene family, member 2; N-CoR, Nuclear receptor co-repressor; NuRD, Nucleosome remodelling and deacetylating; RbAp46, Retinoblastoma associated protein of 46 kDa; SAP18, Sin3 associated protein of 18kDa; SDS3, Suppressor of defective silencing 3; Sin3, Switch insensitive 3; SMRT, Silencing mediator for retinoid and thyroid receptors; ZNF217, Zn finger factor 217 kDa.

HDACs are divided into four classes: I (HDAC1, -2, -3, and -8), II (HDAC4, -5, -6, -7, -9, and -10), III (Sirt1, -2, -3, -4, -5, -6, and -7) and IV (HDAC11) [41-43]. The widely expressed class I HDACs are exclusively localized to the nucleus whereas the more restricted class II HDACs shuttle between the nucleus and cytoplasm (Table 2). There is evidence that these different HDACs target different patterns of acetylation and regulate different genes [40]. The different HDACs are also likely to be regulated differently. HDACs interact with corepressor molecules, such as nuclear receptor corepressor (NCoR), ligand-dependent corepressor (LCoR), NuRD (nucleosomes remodelling and decatylase) and mSin3 (Switch independent 3), all of which aid HDACs in gene repression and may provide specificity by selecting which genes are switched off by HDAC [41,44,45] (Figure 5).

**Table 2:** HAT and HDAC family members

**HDAC families**	**Substrate**
**Class I (Rpd3 homologs)**	
HDAC 1	H2A, 2B, 3, 4, AR, ER, SHP, YY1
HDAC 2	H2A, 2B, 3, 4, GR, YY1
HDAC 3	H2A, 2B, 3, 4, GR, SHP, GATA1, YY1
HDAC 8	H2A, 2B, 3, 4
**Class II (Hda1 homologs)**	
HDAC 4	H2A, 2B, 3, 4, GATA1
HDAC 5	H2A, 2B, 3, 4, GATA1
HDAC 6	H2A, 2B, 3, 4, tubulin, SHP
HDAC 7	H2A, 2B, 3, 4
HDAC 9	H2A, 2B, 3, 4
HDAC 10	H2A, 2B, 3, 4
**Class III (Sir2 homologs)**	
SIRT 1	
SIRT 2	
SIRT 3	
SIRT 4	Non-histone proteins
SIRT 5	e.g. tubulin, p65, p53
SIRT 6	
SIRT 7	
**Class IV (Rpd3 homolog)**	
HDAC 11	H2A/H2B/H3/H4
**HAT families**	
**GNATs (Gcn5-related acetyltransferase)**	
Hat1	H4/H2A
Gcn5 and Gcn5L	H3 K9/K14/H2B, c-Myc
Elp3	H3/H4
Hpa2	H3/H4
PCAF	H3/H4, c-Myc, GATA2
**MYST (MOZ, Ybf2/Sas3, Sas2, Tip60-related)**	
Esa1	H4/H2A
Tip60	H4/H2A, c-Myc, AR
MOF	H4 K16/H3/H2A
MOZ	
Sas3	H3/H4
Sas2	H4K16
**P300/CBP**	
P300/CBP	H2A/H2B/H3/H4, p53, p65, AR, ER
**General transcription factor HATs**	
TAF250	H3/H4
TFIIIC	H2A/H3/H4
**Nuclear hormone related HATs**	
SRC1	H3/H4
SRC3/ACTR	H3/H4

For abbreviations used see text.

The activities of most if not all HDACs are regulated by protein-protein interactions. In addition, many HDACs are regulated by post-translational modifications as well as by subcellular localization. HDACs generally exist as a component of stable large multi-subunit complexes, and most, if not all, HDACs interact with other cellular proteins. With the exception of mammalian HDAC8, most purified recombinant HDACs are enzymatically inactive [46]. Any protein that associates with HDACs, therefore, has the potential to activate or inhibit the enzymatic activity of HDACs. Likewise, HDACs, in general, have no DNA binding activity, therefore, any DNA-binding protein that targets HDACs to DNA or to histones potentially can affect HDAC function.

Human HDAC1 and HDAC2 exist together in at least three distinct multi-protein complexes called the Sin3, the NuRD, and the Co-repressor of REST (RE1 silencing transcription factor, CoREST) complexes [46](Figure 5). Sin3 and NuRD complexes share a core comprised of four proteins: HDAC1, HDAC2, retinoblastoma associated protein (RbAp)46, and RbAp48. In addition, each complex contains unique polypeptides (Sin3, sin3 associated protein (SAP)18, and SAP30 in the Sin3 complex; Mi2, metastasis-associated gene family (MTA)-2, and methyl CpG binding domain (MBD)3 in the NuRD complex) which are essential for HDAC activity and function [47,48]. Thus the NuRD complex may link acetylation and methylation in the regulation of gene expression [46]. Similar results are seen for HDAC activity within the CoREST complex [49]. Furthermore, HDAC3 activity is dependent upon silencing mediator of retinoid and thyroid receptor (SMRT) and nuclear receptor corepressor (N-CoR) association [46].

Unlike HDAC3, the class II HDACs cannot be activated by SMRT/N-CoR alone. Instead, the enzymatic activity of HDAC4, 5, and 7 is dependent on the association with the HDAC3/SMRT/N-CoR complex [46]. These studies suggest that HDAC4, 5, and 7 are not active deacetylases but recruit preexisting enzymatically active SMRT/N-CoR complexes containing HDAC3 [50] (Figure 5).

All mammalian HDACs possess potential phosphorylation sites and many of them have been found to be phosphorylated *in vitro *and *in vivo*. HDAC1 phosphorylation may either alter its conformation into a more favourable enzymatic active form or affect the ability of HDAC1 to interact with proteins, such as MTA2 and SDS3, which can subsequently stimulate its activity and consequently enhance its enzymatic activity [46]. Similarly, HDAC2 phosphorylation is necessary for both enzymatic activity and association with the corepressors mSin3 and Mi2 [46]. The activity of class II HDACs may also be regulated by phosphorylation via modulating their subcellular localization [46]. HDACs must reside in the nucleus in order to deacetylate histones and to repress transcription, therefore, signals that enhance HDAC nuclear localization must affect HDAC activity. HDAC1, 2, and 8 are predominantly nuclear proteins but in contrast, HDAC3 can be found both in the nucleus and cytoplasm and the nuclear/cytoplasmic ratio depends on cell types and stimuli [46]. Thus, in response to IL-1β stimulation, the N-CoR/TAB2/HDAC3 corepressor complex undergoes nuclear to cytoplasmic translocation, resulting in derepression of a specific subset of NF-κB-regulated genes [51].

In contrast, experiments in cardiac myocytes shows that class II HDACs shuttle between the nucleus and the cytoplasm where they associate with 14-3-3 proteins [52,53]. The binding of class II HDACs to 14-3-3 is absolutely dependent on phosphorylation of conserved N-terminal serine residues and this association results in sequestration of HDACs to the cytoplasm [52,53]. Furthermore, CaMK-mediated phosphorylation of HDACs 4, 5, 7, and 9 promotes their association with 14-3-3 proteins resulting in increased retention of HDACs in the cytoplasm. Binding of 14-3-3 has been suggested to mask an N-terminal nuclear localization signal [52,53].

Interestingly, HDACs can autoregulate their own expression by feedback mechanisms utilising the DNA binding actions of transcription factors such as NF-Y (nuclear factor Y) and Sp1. Furthermore, some degree of cross-talk in this regulation must also occur as changes in HDAC1 expression can also affect the expression of other class I HDACs [46]. Recent evidence [54] has shown that nitration of HDAC2 can lead to protein degradation. Proteasomal degradation appears to be a major mechanism of regulation of HDAC function [46].

### Histone methylation

Histone methylation has been implicated for over 40 years in the control of gene expression [26]. Histones may be methylated on either lysine (K) or arginine (R) residues. Due to their small size and their charged nature it is unlikely that these marks alter chromatin structure. It is therefore believed that methylation of K or R residues forms a binding site or interacting domain allowing other regulatory proteins to be recruited. Methyl-K residues may exist in either the mono-, di- or tri-methylated forms. In contrast, R methylation may be either mono-methylated or di-methylated although a further complexity is added by the ability of di-Me-R to be symmetrical or asymmetrical [30]. Currently, there are at least 17 K and 7 R residues known to be methylated suggesting a large number of possible combinations.

Most of our knowledge concerning the role of methylation in gene expression has come from experiments in yeast and *Drosophila *however, general principles appear to hold true in man [30]. Histone H3 and H4 methylation has been most studied and distinct forms are presence within heterochromatin (condensed, heritable and transcriptionally inert chromatin) and euchromatin (loosely packed and transcriptionally active chromatin). Thus methylated forms of H3K9, H3K27, H3K79 and H4K20 are found to be associated with heterochromatin whereas activated genes with euchromatin are associated with methylated H3K4 and H3K36 histones. Upon selective gene activation further methylation of these histones (H3K4 & H3K36) within the 5' controlling regions of genes occurs [30]. These posttranslational modifications are carried out by histone methyl-transferases (HMT), which covalently modify lysines and arginines on histones. These modifications, in combination with acetylations, are thought to inscribe a histone pattern that recruits factors that affect transcription [55].

The discovery that one of the well-studied Su(var) genes encoded a histone methyltransferase (HMT) was a major breakthrough in the understanding the function of H3K-methylation [1]. The *Drosophila *Su(var)3–9 gene was originally pulled out of a genetic screen for transcriptional silencing associated with heterochromatin [56]. Subsequently, the human homolog, Suv39H1, was shown to specifically methylate histone H3 at K9 [57]. Structure-function analyses of Suv39H1 and other HMTs indicated that the SET domain was responsible for HMT activity. The highly conserved SET domain is named after three proteins all with silencing properties: Su(var)3–9, enhancer of zeste [E(Z)], and trithorax (TRX) [56]. Many SET domain-containing proteins have high specificity for different sites on H3 and H4 but it is important to note that not all SET domain-containing proteins are HMTs, nor are the activities of all HMTs mediated by SET domains [1]. For example, Dot1p is a non-SET domain-containing enzyme that methylates H3 at Lys79 [1,58].

As with acetylation, the functional consequence of histone K methylation depends upon the proteins that recognize the particular modification. Protein that induce gene repression, such as heterochromatin protein 1 (HP1) (Figure 3) or the *Drosophila *Polycomb (PC) protein, contain a chromodomain that allows them to specifically recognize the appropriate repressive methylation mark (H3K9 and H3K27 respectively) [30], whereas the activating protein chromodomain helicase DNA-binding protein 1 (CHD1) from *Saccharomyces cerevisiae *uses its chromodomain to bind the activating methylated H3K4 [59]. Other domains, important for the recognition of distinct methylated lysine residues have also evolved e.g. for the recruitment of proteins involved in DNA repair (see later) although it is not known generally how recruitment of distinct proteins to particular methylated lysines leads to the desired functional effect [30].

### Demethylation of lysines

The enzyme LSD1 (lysine-specific demethylase 1) which is able to demethylate H3K4 has recently been identified [60]. The ability to target the activating methylated H3K4 site correlates with its expression in a number of repressor complexes [30]. However, LSD1 can only demethylate the mono- or di-methylated forms of H3K4 despite the fact that the tri-methylated state is most closely associated with active genes. This suggests that other enzymes must exist although the action of co-factors may also be important. In addition, it has been reported that the androgen receptor may be able to alter the specificity of LSD1 from H3K4 to H3K9, and thereby converts the demethylase from a repressor to an activator of transcription [61]. This data is controversial and requires confirmation. The recent discovery of demethylases has opened up a new area of research and suggested that methyl marks are not necessarily permanent. This agrees with evidence from stem cells and cell lines indicates that patterns of gene expression thought to be under epigenetic control can be reversed [2,62].

### Arginine methylation and demethylation

There are a number of protein arginine methyltransferases (PRMTs) and R methylation is only found on chromatin when genes are actively transcribed particularly in response to oestrogen receptor activation although a methyl R binding protein has not been reported [63]. Interestingly, during oestrogen-mediated gene induction, H3R2 methylation appears to be transient or even cyclical [64] which suggest the existence of enzymes that reverse R methylation. Recently, an enzyme peptidyl arginine deiminase 4 (PADI4) has been found which removes the methyl group mono-methyl R residues in H3 and H4 [65,66]. PAD14 converts the R residue to citrulline but whether citrulline can be removed or converted back to R is unknown as is the answer to the question as to whether citrulline itself can act as an epigenetic mark. Interestingly, PAD14 activity is linked to the repression of an oestrogen-controlled gene, *pS2 *[30].

### Cross-talk between histone marks

Cross-talk between different histone marks can also have a profound effect on enzyme activity [1]. For instance, ubiquitylation of H2B K123 by the E2 ubiquitin conjugating enzyme Rad6 is required for subsequent di-methylation of H3 K4 by Set1p or H3 K79 by Dot1p [38]. Prior histone marks can also inhibit subsequent modifications [1]. For example, H3 S10 phosphorylation inhibits subsequent H3 K9 methylation, and of course H3 K9 methylation can also block acetylation of this same residue. More recently it has been demonstrated that S10 phosphorylation by Aurora B kinase can lead to the dissociation of HP1 from heterochromatin without affecting K9 methylation status [67,68]. An excellent example of even more complex cross-talk is exemplified during p53-dependent transcriptional activation *in vitro *[69]. In this case methylation of H4 R3 by PRMT1 stimulates CBP-p300 acetylation of H4 K5, K8, K12 and K16, which in turn promotes the methylation of H3 R2, R17 and R26 by another PRMT family member, CARM1. Thus, positive and negative crosstalk ultimately generates the complex patterns of gene or locus-specific histone marks associated with distinct chromatin states.

### Histone variants

Chromatin arrays also contain novel types of nucleosome that harbour one or more variant isoforms of the core histones [1]. For instance, nucleosomes assembled at yeast and mammalian centromeres contain a histone H3 variant, Cse4/CENP-A, which is essential for centromere function or assembly. Another histone H3 variant, H3.3, replaces canonical histone H3 during transcription, generating a mark of the transcription event [1]. Several variants of histone H2A have also been identified. The macro-H2A variant is restricted to metazoans and functions in X chromosome inactivation, while H2AZ (also known as H2A.F/Z or H2AvD) is found in all eukaryotes. Surprisingly, H2AZ is required for one or more essential roles in chromatin structure that cannot be replaced by *bona fide *histone H2A [70]. In most cases, it is not known how histone variants alter nucleosome structure or change the folding properties of nucleosomal arrays [70]. Once a histone variant is targeted to a specific locus, there is the potential for creation of novel chromatin domains that have distinct regulatory properties. For instance, the amino-terminal tail of CENP-A lacks the phosphorylation and acetylation sites that are normally modified in histone H3 at transcriptionally active regions [71].

### Methylation and RNA interference (RNAi)

DNA methylation has long been shown to have a transcriptional silencing function which may reflect the fact that several HDAC-containing complexes possess methyl-DNA binding motifs [1]. Furthermore, Suv39H1/2 knockout cells from mice have an abnormal pericentric heterochromatin DNA methylation pattern [72]. Mutually reinforcing relationships between histone modifications and DNA methylation have been found such as H3-K9 methylation is a prerequisite for DNA methylation and DNA methylation can also trigger H3-K9 methylation [1,3,73]. It is likely that both DNA and histone methylation pathways leave epigenetic marks that are required for stable and long-term epigenetic silencing. However, it is unclear what initiates the recruitment of the different epigenetic modifiers to their specific target sequences [1,3].

Since its discovery in 1990 as a means of controlling Petunia colour [74] and the more recent demonstration in mammalian cells there has been great interest in the mechanisms by which RNA interference (RNAi) controls mitotically heritable transcriptional silencing [75,76]. It is clear that components of the RNAi machinery can exist in complexes with the chromodomain protein CHP1 which may enable targeting to specific methyl K residues [75,76]. In addition, deletion of components of the RNAi machinery results in impaired centromere function, a derepression of transgenes integrated at centromeres, and a loss of the characteristic H3-K9 methylation and HP1 association [75,76]. Furthermore, miRNAs and antisense RNAs are involved in the silencing of some mammalian imprinted genes [77] and in dosage compensation in mammals [75,76] suggesting that RNA is able to direct histone modifications (for example, H3-K9 methylation) and DNA methylation to specific loci, thereby evoking heritable and stable silencing [75,76]. Finally, there is a report of a case of α-thalassaemia showing how antisense transcription could lead to DNA methylation and stable silencing of the HBA2 globin gene [78].

### Inheritance of epigenetic marks on histones

**Figure 6:**
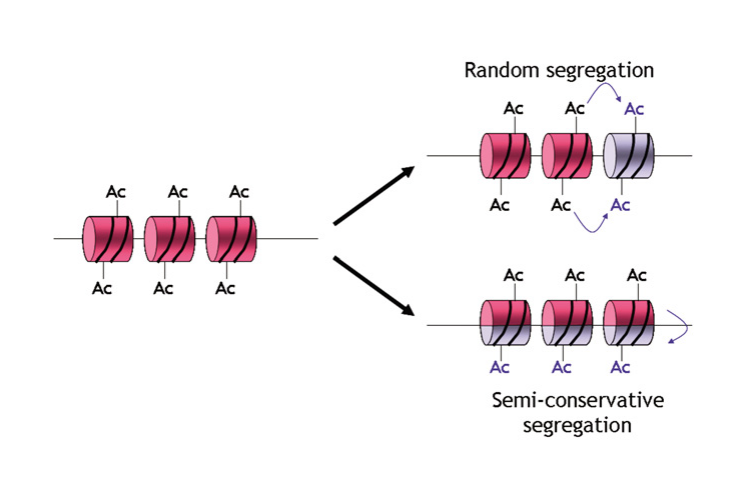
During replication, parental nucleosomes are recycled and deposited onto the two daughter strands. In the random segregation model, the parent histone octamers (*red cylinders*) are intact and pass to the two daughter strands in a random manner. Newly assembled nucleosomes (*grey cylinders*) fill in the gaps not occupied by the parental octamers and histone-modifying enzymes copy the parental histone modifications (e.g. Acetylated groups, Ac) to newly assembled nucleosomes. In the semi-conservative model, the parent histone octamers split in half and are equally distributed among the two daughter strands (*red halves*). Nucleosome assembly complexes then deposit newly synthesized histones to complement the existing halves of the nucleosomes (*grey halves*) present on the daughter strands. In this case, histone modifying enzymes would copy the modifications (e.g. Ac) to the new half of the nucleosomes from the old half (symbolized by the *green arrows*).

Little detail concerning the mechanisms for inheritance of histone modifications is known in contrast to that for the inheritance of DNA methylation through mitotic cell division [1]. Methylated K residues do not have a rapid turnover rate and early studies looking at the turnover rate of histone methylation found that the half-life of the methyl mark on histones was equal to that of the protein itself indicating an irreversible modification that persisted through cell division [79]. In addition, even the highly dynamic acetyl K modifications are maintained during mitosis and inheritance of acetylation patterns may be essential to maintain gene expression profiles through successive generations [80]. Thus, successful propagation of histone modification patterns requires a way of copying/replicating preexisting modifications onto the newly assembled nucleosomes [1]. During DNA replication, preexisting nucleosomes of the parental genome are recycled and deposited onto the newly generated daughter strands, and therefore, any stable histone modifications can potentially be transferred from one generation to the next [1] (Figure 6).

Parental nuclesomes may divide in a semiconservative manner whereby the parental histone octamer is split into H2A-H2B/H3-H4 heterodimers that are then equally segregated onto the two daughter DNA strands [81]. The nucleosome assembly complex then deposits newly synthesized histones to complete the preexisting half of the nucleosomes raising the potential to faithfully and equally transmit histone-associated information from parent to daughter DNA strands [1,81]. In the DNA methylation process, copying of the methylation pattern during replication is mediated by DNMT1 that preferentially methylates hemimethylated DNA [1]. A similar mechanism could be invoked for HMTs and HATs whereby recruitment to selectively modified histone residues may be afforded by the use of chromo- and bromo-domains within the enzymes themselves.

### Role of epigenetics in DNA damage/repair

Following a double stranded strand break (DSB) DNA repair processes such as homologous recombination and single-strand annealing occur and the chromatin adjacent to this DSB plays a role in the repair and signalling events. Phosphorylation of the C terminus of histone H2AX (a variant of histone H2A) is an early event following DNA damage induced by ionizing radiation or by HO endonuclease activity. This is a result of the action of two related PI3K-like kinases called ATR and ATM [82,83]. Phosphorylation of H2AX forms a binding interface that allows recruitment of cohesions or adaptor proteins to the site of DSB and subsequent recruitment of the repair machinery [82,83].

Chromatin remodeling complexes such as NuA4 are also recruited to DSB via proximal H2AX [83,84] possibly allowing the access to or processing of DNA by repair proteins. Interestingly, NuA4 also contains histone acetyltransferase activity and can acetylate histone H4, which is important for resistance to DNA-damaging agents [84]. Importantly, abrogation of NuA4 function sensitizes cells to DSB-inducing agents [83,84].

Other histone modifications such as ubiquitination, acetylation, and methylation have also been implicated in the DNA damage checkpoint and repair pathways [82,83]. Despite bulk histone methylation not changing after DNA damage [85] histone methylation does appear to contribute to the repair process directly interacting with checkpoint adaptor proteins. For example, in mammals, H3-K79-Me is important for localization of the adaptor protein 53BP1 [85] and cells deficient in Dot1, the HMT responsible for lysine 79 methylation, are unable to form 53BP1 foci after DNA damage. However, the process is more complex as neither chromatin remodelling complexes nor histone modifications are absolutely required for adaptor proteins to function in the repair of DSB due to ionizing radiation [82,83].

### Epigenetic diseases

Heritable patterns of gene silencing are essential to maintain normal development and cell differentiation in man. Many inherited or somatically acquired diseases which involve learning disorders are associated with chromosomal alterations [3]. Examples of these include mutations in the *ATRX *gene which results in consistent changes in the pattern of methylation of ribosomal DNA, and fragile X syndrome which occurs when a CGG repeat in the *FMR1 *5' untranslated region expands and becomes methylated [3], causing the gene to be silenced and creating a visible 'fragile' site on the X chromosome. The gross chromosomal anomalies seen in these diseases points to a central role for epigenetic mechanisms in chromosome architecture. Furthermore, mutations in the *DNMT3b *gene causes ICF (immunodeficiency, centromeric region instability and facial anomalies) syndrome [14,86].

A number of the features of complex diseases that are not explained by genetics may be explained, at least in part, by the inheritability, partial stability and reversibility of epigenetic regulation [87]. Epigenetic regulation has been proposed to account for age-of-onset effects, sex effects, parent-of-origin effects (which are very important in asthma and COPD), disease fluctuations and might provide an explanation for the phenotypic discordance often observed among monozygotic twins (70% for multiple sclerosis, 30% to 50% for diabetes, and 25% for asthma) [87,88]. Interestingly, PADI4 polymorphisms have been associated with rheumatoid arthritis in some, but not all, populations [89,90]. More recently, global histone modification patterns have been shown to predict the risk of prostate cancer recurrence [91].

### Epigenetic control of inflammatory gene expression in lung and airway cells

#### Induction of inflammatory genes by nuclear factor κB (NF-κB)

Although numerous different pathways are activated during the inflammatory response, nuclear factor kappaB (NF-κB) is thought to be of paramount importance in asthmatic inflammation because it is activated by numerous extracellular stimuli including cytokines, such as tumour necrosis factor-α (TNFα) and interleukin-1β (IL-1β), viruses and immune challenges [92]. In addition, it is a major target for glucocorticoids [93]. NF-κB is ubiquitously expressed within cells, and it not only controls induction of inflammatory genes in its own right but also enhances the activity of other cell- and signal-specific transcription factors [92,94]. Activation of NF-κB allows it to translocate into the nucleus where it associates with sequence specific DNA binding elements in the promoter region of responsive genes [95].

NF-κB can induce histone acetylation and other histone modifications in a temporal manner [96,97] leading to recruitment of other co-activator and remodelling complexes and the induction of inflammatory gene expression [95]. NF-κB-induced acetylation occurs preferentially on histone H4, rather than histones 2A, 2B or 3, in epithelial cells and is directed primarily towards lysine residues 8 and 12 at NF-κB responsive regulatory elements [96]. It is not known whether modifications on H2A and H2B occur after NF-κB association/activation. The "histone code" would suggest that even small changes in histone tail modifications could have marked structural changes and allow recruitment of distinct co-activator complexes. Upon DNA binding, NF-κB recruits a large co-activator complex that contains the HAT proteins cAMP response element binding protein (CREB) binding protein (CBP) and p300/CBP (PCAF), although neither of these are the major HAT activated by NF-κB [96]. Several other HATs have been reported to be associated with NF-κB, including transcriptional intermediary factor-2 (TIF-2), also known as glucocorticoid receptor interacting protein-1 (GRIP)-1; p300; and members of the p160 family and steroid receptor coactivator-1 (SRC-1) [98].

Recently it has become apparent that NF-κB activated by distinct cellular stimuli controls the expression of different patterns of genes [99-101] due to differing temporal profiles of NF-κB activation and nuclear retention. These results suggest that subtle alterations in NF-κB activation conditions may have marked effects on co-factor/remodelling complex recruitment and subsequent gene induction. Furthermore, it has also become clear that small changes in the consensus κB binding site and surrounding bases can have profound effects on the subsequent ability of activated NF-κB to activate gene expression [102]. NF-κB is predominantly composed of the p50/p65 heterodimer [92] and subtle changes in p65 phosphorylation are also influential in regulating NF-κB activity; for example, inactive p65 is nonphosphorylated and is associated predominantly with HDAC1, whereas p65 is phosphorylated following IKK-2 stimulation and is able to bind to coactivator molecules such as p300/CBP [103].

The histone deacetylase inhibitor trichostatin A has been reported to enhance NF-κB-driven inflammatory gene transcription in a number of cell lines [96,103\ensash 105]. Two major mechanisms for this effect have been proposed. In the first case it has been reported that NF-κB has an associated HDAC when bound to DNA that acts as a break on the ability of NF-κB to activate local HAT activity. Inhibition of this associated HDAC leads to increased local HAT activity and elevated inflammatory gene transcription [96,103-105]. Warner Greene and colleagues [105] have proposed an alternative mechanism whereby HDAC3 modifies NF-κB nuclear-cytoplasmic shuttling and association with IκBα resulting in enhance nuclear retention of activated p65 that is insensitive to inactivation by IκBα. More recently using overexpression systems it has been suggested that IκBα can sequester HDAC1 and HDAC3 in the cytoplasm enhancing NF-κB activity [106].

#### Suppression of NF-κB by glucocorticoids

Glucocorticoids are 21-carbon steroid hormones [107] which are thought to freely diffuse from the circulation into cells across the cell membrane and bind to a cytoplasmic receptor (GR). Once the GR is activated, it translocates to the nucleus, binds to specific DNA sequences in the promoter regions of responsive genes and in a process analogous to that seen with NF-κB above recruits a number of coactivators proteins including CBP and SRC-1 to produce a DNA-protein structure that allows enhanced gene transcription [108]. However, despite the ability of glucocorticoids to induce the transcription of anti-inflammatory genes, such as annexin-1, IL-10, and the inhibitor of NF-κB, IκBα, the major anti-inflammatory effects of glucocorticoids are through repression of inflammatory and immune genes induced by NF-κB [109]. This interaction does not appear to alter DNA binding *per se*, thus treatment of asthmatic patients with high doses of inhaled corticosteroids that suppress airway inflammation is not associated with any reduction in NF-κB binding to DNA [110].

The interaction between NF-κB and GR may result in differing effects on histone modifications such as acetylation/deacetylation at the activated p65 transcriptional complex, either through GR binding to, or recruiting, nuclear receptor corepressors such as NCoR, or interestingly under some conditions by the co-activator GRIP-1, and HDACs [96,111] or direct repression of NF-κB- associated HAT activity [96] resulting in alterations in histone modifications at the GM-CSF promoter in epithelial cells [96] resulting in attenuation of gene expression. Similar data has also been reported in primary airway smooth muscle cells where fluticasone was able to attenuate TNFα-induced p65 association with the native CCL11 promoter and block TNFα-induced histone H4 acetylation [112].

Using chromatin immunoprecipitation (ChIP) assays we have demonstrated that corticosteroids reverse the acetylation of the promoter of inflammatory genes such as GM-CSF [96]. Other genes are not recognised through this mechanism, so corticosteroids do not switch off genes involved in basal cell functions, proliferation or survival. Furthermore this explains why corticosteroids are relatively safe, as side effects may be mediated mainly by gene activation mechanisms, which requite higher concentrations of corticosteroids, rather than via gene repression and HDAC recruitment.

It has become clear that histones are not the only targets for histone acetylases and recent evidence has suggested that acetylation of transcription factors can modify their activity. For example, the p65 component of NF-κB can also be acetylated thus modifying its transcriptional activity [113]. We have recently reported that GR is also acetylated upon ligand binding at K494 and K495 and that deaceylation by HDAC2 is critical for interaction with p65, at least at low concentrations of dexamethasone, without affecting the ability of GR to associate with GREs [113]. Furthermore, specific knockdown of HDAC2 by RNA interference resulted in reduced sensitivity to dexamethasone suppression of IL-1β-stimulated GM-CSF release and prevented p65 association with GR. In addition, site -directed mutagenesis of K494 and K495 reduced GR acetylation and the ability to repress NF-κB-dependent gene expression became insensitive to TSA. Finally, we have shown that overexpression of HDAC2 in glucocorticoid insensitive alveolar macrophages from patients with COPD is able to restore glucocorticoid sensitivity [113]. This data suggests that reduction of HDAC2 plays a critical role in glucocorticoid insensitivity in repressing NF-κB, but not GRE, -mediated gene expression.

### HATS/HDACs in airway diseases

Little or no data is available about epigenetic marks in respiratory diseases probably due to a lack of research rather than a lack of epigenetic marks. In bronchial biopsies from patients with asthma there is a marked increase in HAT and a small reduction in HDAC activity compared to normal airways [114] (Table 3). Similar changes are found in alveolar macrophages obtained by bronchoalveolar lavage from patients with asthma [115]. Changes in activity are associated with reductions in select proteins e.g. there is a small reduction in the expression of HDAC1, but expression of HDAC2 and 3 is normal in these cells. Peripheral blood mononuclear cells (lymphocytes and monocytes) appear to have normal HAT and HDAC activity, indicating that these changes occur locally in the airways of asthmatic patients. Interestingly, in patients with asthma who smoke there is a significantly greater reduction of HDAC activity in bronchial biopsies than in non-smoking asthmatic patients (unpublished observations) and this may account for why these smoking asthmatics have more severe asthma and perhaps a relative steroid insensitivity [116].

**Table 3:** Changes in HAT and HDAC activity and protein expression in asthma and COPD.

	HDAC activity	HDAC 1	HDAC 2	HDAC 5	HDAC 8	HDAC 3	HAT activity
Asthma	↓	↓	↓	ND	ND	ND	↑
Smoking	↓	-	↓	-	-	-	-
COPD	↓	-	↓^1^	↓^1^	↓^1^	↓^2^	-

^1^mRNA expression also decreased compared with healthy non-smokers ^2^mRNA expression also decreased compared with healthy smokers

In contrast to asthma, in COPD there is no change in HAT activity but a marked reduction in HDAC activity in the lung parenchyma and this decrease is correlated with disease severity [117]. The reduction in HDACs in peripheral lung and BAL macrophages is selective with a marked reduction in HDAC2, with lesser reduction in HDAC5 and HDAC8 expression, but normal expression of the other class 1 and 2 HDACs. Furthermore, HDAC5 expression is predominantly cytoplasmic rather than nuclear in patients with COPD. The reduction in HDAC activity is also related to the intensity of inflammation, as measured by expression of IL-8 and the number of inflammatory cells in small airways [118]. In addition, the lack of clinical efficacy of corticosteroids in COPD compared with marked effects in asthma [119-121] may be explained, at least in part, by an inhibitory effect of cigarette smoking and oxidative stress on HDAC function [122]. Asthmatic patients who smoke have more severe disease and are also resistant to the anti-inflammatory effects of corticosteroids [116,123]. Alveolar macrophages from normal smokers show a reduction in HDAC activity and expression of HDAC2 (Table 3) and this is correlated with an increase in release of TNF-α and IL-8 in response to an inflammatory stimulus Similarly, in smoking rats there is a reduced expression and activity of HDAC2 which is associated with increased inflammatory gene expression and reduced corticosteroid sensitivity [124].

**Figure 7:**
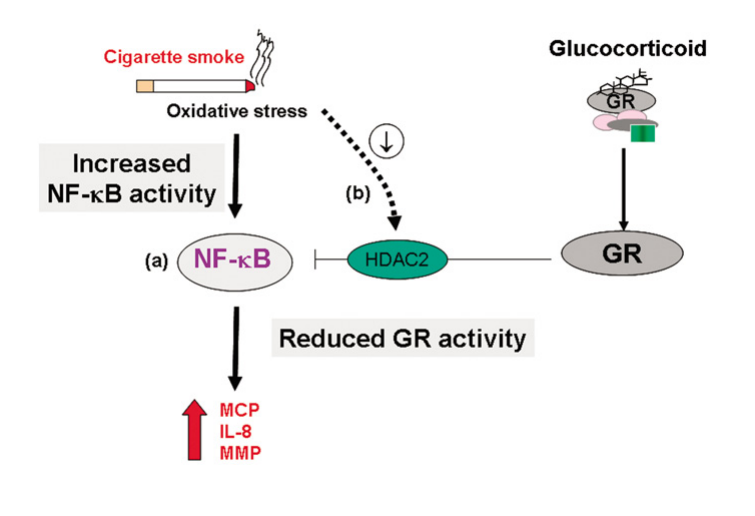
Oxidative stress can increase the expression inflammatory genes such as interleukin (IL)-8, matrix metalloproteinase (MMP) and monocyte chemotactic protein (MCP) by up-regulating NF-κB activity (a). Oxidative stress can also prevent glucocorticoid receptor (GR) function by (b) suppression of GR-associated histone deacetylase 2 (HDAC2) activity and expression since HDAC2 is recruited by GR to NF-κB to switch off inflammatory gene expression.

In addition to subjects with COPD, patients with asthma who smoke cigarettes also show resistance to the anti-inflammatory actions of corticosteroids and this persists to some extent even in ex-smokers [116,123]. Cigarette smoking is an oxidative stress and may affect several aspects of steroid function including effects on nuclear cofactors [125,126]. Importantly, these effects are reversed by antioxidants [125-127]. Intriguingly, there is also a marked increase in oxidative stress in severe glucocorticoid insensitive asthma [128,129] which also shows reduced HDAC2 expression [115]. This suggests that anti-oxidants or NOS inhibitors, which would reduce the formation of peroxynitrite, may therefore be effective therapies and restore glucocorticoid responsiveness in COPD, severe asthma and asthmatic subjects who smoke (Figure 7).

Recent evidence also suggests that changes in DNA hypersensitivity related to altered methylation patterns and to histone acetylation can occur at the IL-10 locus during T-cell differentiation into Th2 cells and in IL-10-producing regulatory T cells enabling optimal IL-10 gene expression [130]. Similar results occur for other Th2 cytokines in human cells [131].

### Virus infections

Adenovirus infection increases the expression of inflammatory genes in epithelial cells *in vitro *and this appears to be mediated via the adenoviral E1A protein, which is capable of interacting with HAT-containing coactivators such as CBP [132]. In COPD lungs there is evidence for latent adenovirus infection and increased expression of E1A protein, so that this may be a mechanism for amplification of inflammation in COPD patients [133,134]. Interestingly, adenovirus infection in guinea pigs amplifies the inflammatory response to allergen [135] and is associated with a significant reduction in HDAC activity in the lungs in ovalbumin-sensitized animals [136]. Persistence of adenovirus infections has also been implicated in steroid-resistance in children with asthma [137]. Other virus infections may also impair the action of HDAC2 and thus induce steroid resistance, but this still needs to be explored. Thus, increased gene transcription in inflammatory diseases may be due to increased HAT, decreased HDAC or a combination of both.

### Lung cancer

Global hypomethylation, dysregulation of DNA methyltransferase and regional hypermethylation in normally unmethylated CpG islands have all been implicated in lung cancer [138]. Specific CpG island methylation seen in the promoter regions of many genes associated with neoplasias such as p16 suggests that abnormal expression or regulation of Dnmt1 may be important in non-small cell lung cancer (NSCLC) [139]. Methylation of the promoter regions in multiple genes has been reported in adenocarcinimas and NSCLC with increasing numbers of methylated genes being associated with tumour progression [140,141]. The ability to detect these changes in peripheral blood and sputum suggest a useful marker for early detection and/or chemoprotective interventions [142].

### Epigenetic therapy

Since many human diseases, in particular cancer, have an epigenetic aetiology investigators have used drugs targeting these processes as novel therapies [3]. Several of these agents including histone deacetylase inhibitors (e.g. SAHA & MS275) and DNA methylase inhibitors (e.g. 5-azacytidine) have been tested in clinical trials with the intention of reactivating the expression of genes that have undergone epigenetic silencing [3]. Results, however, have not proved as successful as predicted from cell line data possibly as a result of cytotoxicity [143]. The prototype methylase inhibitors, 5-azacytidine (5-aza-CR) and 5-aza-2'-deoxycytidine (5-aza-CdR) are converted to the deoxynucleotide triphosphates and are then incorporated in place of cytosine into replicating DNA. They are therefore active only in S-phase cells, where they serve as powerful mechanism-based inhibitors of DNA methylation [3]. DNA methyltransferases get trapped on DNA containing these modified bases (e.g. azacytosine) resulting in the formation of heritably demethylated DNA [144]. However, covalent attachment of the various DMTs to modified DNA may also be responsible for the cytotoxic effects seen severely limiting their utility [3]. In addition, it is no means that that the therapeutic mechanism of action of DNA methylation and HDAC inhibitors is through epigenetic effects. HDAC inhibitors and DNA methylation inhibitors are cytotoxic agents and induce cell-cycle arrest and apoptosis by upregulating p21 and/or p53 [3,145]. Furthermore, loss of genomic methylation causes p53-dependent apoptosis, and p53 represses DNMT1, suggesting a feedback loop between the two proteins [3,146]. Clinical trials with antisense oligonucleotides that target the DNA methyltransferases are also underway [147].

We have shown that the anti-inflammatory effects of theophylline with respect to resduced eosinophilia in bronchial biopsies from asthmatic patients may be mediated via activation of HDAC and that this effect is independent of PDE inhibition [148]. Theophylline appears to preferentially activate class I HDACs, including HDAC2 [149]. However, the exact mechanism whereby theophylline activates HDAC is not yet certain, but is likely to be through signal transduction pathways, probably kinases, that regulate HDAC activity or co-factor association. The effects of theophylline on HDAC appear to be enhanced under conditions of oxidative stress, making it more efficient as a regulator of inflammatory genes [149]. This means that the dose of theophylline does not have to be increased as the disease becomes more severe as the increase in oxidative stress would increase drug activity.

This predicts that theophylline will enhance the anti-inflammatory actions of corticosteroids and therapeutic concentrations of theophylline markedly potentiate the anti-inflammatory effects of corticosteroids *in vitro *[149]. This may explain why adding a low dose of theophylline is more effective than increasing the dose of inhaled corticosteroids in patients who are not adequately controlled [150-152].

## Summary & conclusions

The recognition that epigenetic modifications of DNA and histones regulate inflammatory gene expression and play a role in diverse functions such as DNA repair, proliferation, RNA interference and in several diseases indicates the global importance of these effects. Their role in lung cancer has become increasingly clear and evidence is accumulating that epigenetic changes may account for some of the heritable effects of cigarette smoking. The reversible nature of these modifications raises the possibility of future therapies directed against these changes.

## List of abbreviations used

53BP1 p53-binding proteinACTR activator of retinoid receptor

AP-1 Activator protein-1

ATM ataxia telangiectasiamutated proteinATR ataxia telangiectasia and Rad3-related proteinATRX α--thalassemia mental retardation X-linked

Brg1 Brahma homolog

CARM1 Co-activator-associated arginine methyltransferase 1

CBP CREB binding protein

CHD1 chromodomain helicase DNA-binding protein 1

Co-REST Co-repressor of REST (RE1 silencing transcription factor)

CREB cAMP response element binding protein

DNMT DNA methyltransferase

Dot1p disruptor of telomeric silencing 1

DSB double stranded strand breakE (var) enhancers of PEV

E(Z) enhancer of zeste

FMR1 Familial Mental Retardation gene 1

FOXO1 Forkhead box protein O1

Gcn5 general control non-derepressible 5

GNAT general control non-derepressible 5 related acetyltransferases

HAT Histone acetyltransferase

Hda1 Histone deacetylase 1 (yeast)

HDAC Histone deacetylase

HMT histone methyl-transferaseHP1 heterochromatin protein 1

ICF immunodeficiency, centromeric region instability and facial anomalies syndrome

LCoR Ligand dependent corepressor

LCoR ligand-dependent corepressor

LSD1 lysine-specific demethylase 1

MBD3 Methyl CpG binding domain 3

Mi2 Mi2 autoantigen

miRNA microRNAMTA-2 Metastasis-associated gene family, member 2

MYST MOZ, Ybf2/Sas3, Sas2 and Tip60

N-CoR Nuclear receptor co-repressor

NF-κB Nuclear factor κB

NSCLC non-small cell lung cancer

NuA4 Nucleosome histone acetyltransferase of histone 4

NuRD Nucleosome remodelling and deacetylating

PADI4 peptidyl arginine deiminase 4

PC *Drosophila *Polycomb

PCAF p300-CBP associated factorPDE Phosphodiesterase

PEV position-effect variegation

PRMT arginine methyltransferase

RbAp46 Retinoblastoma associated protein of 46 kDa

Rpd3 Reduced potassium dependency 3

SAGA Spt-Ada-Gen5-acetyltransferase

SAHA Suberoylanilide hydroxamic acid

SAP18 Sin3 associated protein of 18kDa

SDS3 Suppressor of defective silencing 3

SET Su(var)3–9, Enhancer-of-zeste, Trithorax

Sin3 Switch insensitive 3

Sir2 Silent information regulator 2

SMRT Silencing mediator for retinoid and thyroid receptors

SRC1 steroid receptor coactivator 1

Su(var) suppressors of PEV

SWI/SNF mating type switching/sucrose non-fermenting

TAF250 TBP-associated factor of 250 kDa

TBP TATA box binding protein

TRX trithorax

## Competing interests

The author(s) declare that they have no competing interests

## Authors' contributions

All authors contributed equally towards the writing of this review.
